# Automatic plankton image classification combining multiple view features via multiple kernel learning

**DOI:** 10.1186/s12859-017-1954-8

**Published:** 2017-12-28

**Authors:** Haiyong Zheng, Ruchen Wang, Zhibin Yu, Nan Wang, Zhaorui Gu, Bing Zheng

**Affiliations:** 1 Department of Electronic Engineering, Ocean University of China, No. 238 Songling Road, Qingdao, 266100 China; 2 College of Information Science and Engineering, Ocean University of China, No. 238 Songling Road, Qingdao, 266100 China

**Keywords:** Plankton classification, Image classification, Multiple view features, Feature selection, Multiple kernel learning

## Abstract

**Background:**

Plankton, including phytoplankton and zooplankton, are the main source of food for organisms in the ocean and form the base of marine food chain. As the fundamental components of marine ecosystems, plankton is very sensitive to environment changes, and the study of plankton abundance and distribution is crucial, in order to understand environment changes and protect marine ecosystems. This study was carried out to develop an extensive applicable plankton classification system with high accuracy for the increasing number of various imaging devices. Literature shows that most plankton image classification systems were limited to only one specific imaging device and a relatively narrow taxonomic scope. The real practical system for automatic plankton classification is even non-existent and this study is partly to fill this gap.

**Results:**

Inspired by the analysis of literature and development of technology, we focused on the requirements of practical application and proposed an automatic system for plankton image classification combining multiple view features via multiple kernel learning (MKL). For one thing, in order to describe the biomorphic characteristics of plankton more completely and comprehensively, we combined general features with robust features, especially by adding features like Inner-Distance Shape Context for morphological representation. For another, we divided all the features into different types from multiple views and feed them to multiple classifiers instead of only one by combining different kernel matrices computed from different types of features optimally via multiple kernel learning. Moreover, we also applied feature selection method to choose the optimal feature subsets from redundant features for satisfying different datasets from different imaging devices. We implemented our proposed classification system on three different datasets across more than 20 categories from phytoplankton to zooplankton. The experimental results validated that our system outperforms state-of-the-art plankton image classification systems in terms of accuracy and robustness.

**Conclusions:**

This study demonstrated automatic plankton image classification system combining multiple view features using multiple kernel learning. The results indicated that multiple view features combined by NLMKL using three kernel functions (linear, polynomial and Gaussian kernel functions) can describe and use information of features better so that achieve a higher classification accuracy.

## Background

Plankton, including phytoplankton and zooplankton, are the main source of food for organisms in the ocean and form the base of marine food chain. As the fundamental components of marine ecosystems, plankton is very sensitive to environment changes, and its abundance plays an important role on the ocean ecological balance. Therefore, the study of plankton abundance and distribution is crucial, in order to understand environment changes and protect marine ecosystems.

In the early days, researchers investigated the distribution and abundance of plankton with traditional techniques, such as Niskin bottles, pumps and towed nets, to collect the samples. And then, the classification and counting were done manually by experts. These traditional methods for the study of plankton are so laborious and time consuming that hindered the understanding process of plankton.

To improve the efficiency, many imaging devices, including *in situ* and in the lab, have been developed for collecting plankton images, such as Video Plankton Recorder (VPR) [1], Underwater Video Profiler (UVP) [2], Shadowed Image Particle Profiling Evaluation Recorder (SIPPER) [3], Zooplankton Visualization System (ZOOVIS) [4], Scripps Plankton Camera (SPC) [5], Imaging FlowCytobot (IFCB) [6], *In Situ* Ichthyoplankton Imaging System (*IS*IIS) [7], ZooScan [8], and so on. These imaging devices are able to generate an enormous amount of plankton images within a short time. However, if these collected images are manually classified and counted, there will be a daunting task. Therefore, automatic classification systems of plankton images are required to address the huge amounts of images [9].

Currently, some systems have been developed for plankton image classification [10]. Imaging *in situ* Tang et al. [11] designed a recognition system combining moment invariants and Fourier descriptor with granulometric features using learning vector quantization neural network to classify plankton images detected by VPR; then Hu and Davis [12] improved the classification system with co-occurrence matrices (COM) as the feature and a Support Vector Machine (SVM) as the classifier. Luo et al. [13, 14] presented a system to recognize underwater plankton images from SIPPER, by combining invariant moments and granulometric features with some specific features (such as size, convex ratio, transparency ratio, etc.), and using active learning in conjunction with SVM; and Tang et al. [15] applied shape descriptors and a normalized multilevel dominant eigenvector estimation (NMDEE) method to select a best feature set for binary plankton image classification; then Zhao et al. [16] improved the binary SIPPER plankton image classification using random subspace. Sosik and Olson [17] developed an approach that relies on extraction of image features, including size, shape, symmetry, and texture characteristics, plus orientation invariant moments, diffraction pattern sampling, and co-occurrence matrix statistics, which are then presented to a feature selection and SVM algorithm for classification of images generated by IFCB. Bi et al. [18] also developed a semi-automated approach to analyze plankton taxa from images acquired by ZOOVIS. Faillettaz et al. [19] post-processed the computer-generated classification for images collected by *IS*IIS using Random Forest (RF) obtained with the ZooProcess and PkID toolchain [8] developed for ZooScan to describe plankton distribution patterns. Imaging in the lab ADIAC [20], which stands for Automatic Diatom Identification And Classification, integrated the shape and texture features with Decision Tree (DT), Neural Network (NN), k Nearest Neighbor (kNN) and ensemble learning methods for diatom recognition [21–23]; Dimitrovski et al. [24] presented a hierarchical multi-label classification (HMC) system for diatom image classification evaluated on the ADIAC [20] database. DiCANN [25] developed a machine learning system for Dinoflagellate Categorisation by Artificial Neural Network. Gorsky et al. [8] presented a semi-automatic approach that entails automated classification of images followed by manual validation within ZooScan integrated system. Bell and Hopcroft [26] assessed ZooImage software with the bundled six classifiers (LDA, RPT, kNN, LVQ, NN, and RF) for the classification of zooplankton. Mosleh et al. [27] developed a freshwater algae classification system by using Artificial Neural Network (ANN) with extracted shape and texture features. Santhi et al. [28] identified algal from microscopic images by applying ANN on extracted and reduced features such as texture, shape, and object boundary. Verikas et al. [29] exploited light and fluorescence microscopic images to extract geometry, shape and texture feature sets which were then selected and used in SVM as well as RF classifiers to distinguish between *Prorocentrum minimum* cells and other objects.

Analysis of the aforementioned methods shows the performance of plankton image classification systems based on applied features and classifiers, among which the general features, such as size, invariant moments, co-occurrence matrix, Fourier descriptor, etc., and the traditional classifiers, such as SVM, RF, ANN, etc., are most commonly used respectively [8, 11–13, 17, 20, 25, 27, 29]. However, these features usually suffer from robustness shortage and cannot represent the biomorphic characteristics of plankton well. Also the traditional classifiers usually have not high prediction accuracy on different datasets especially more than 20 categories so that they are hard to be directly applied for ecological studies [8, 18, 19]. Recently, with the development of computer vision technologies, some image features (descriptors) have been developed, such as Histograms of Oriented Gradients (HOG), Scale-Invariant Feature Transform (SIFT), Shape Context (SC), Local Binary Pattern (LBP), etc., and they have been proven to be robust against occlusion and clutter, also have a good performance on object detection, recognition and classification [30]. Thus, we think that it’s the time to apply these new robust image descriptors to represent the characteristics of plankton for better classification performance.

In addition, the morphological characteristics of plankton can be described from different views with diverse features, such as shape, gray, texture, etc. [17, 27]. However, directly concatenating all the features into one that is fed to a single learner doesn’t guarantee an optimum performance [31], and it may exacerbate the “curse of dimensionality” [32]. Therefore, we consider that multiple kernel learning (MKL), where different features are fed to different classifiers, might be helpful and necessary to make better use of the information and improve the plankton image classification performance.

Furthermore, the literature of plankton image classification shows that most methods are developed for the specific imaging device and only address a relatively narrow taxonomic scope. Nevertheless, for the abundant species in a wide taxonomic scope from phytoplankton to zooplankton located in all over the world [33], it’s really impossible to design one specific classification system for each application.

In this paper, inspired by the analysis of literature and development of technology, we focus on the requirements of practical application and propose an automatic system for plankton image classification combining multiple view features via multiple kernel learning. On one thing, in order to describe the biomorphic characteristics of plankton more completely and comprehensively, we combine the general features with the latest robust features, especially by adding features like Inner-Distance Shape Context (IDSC) for morphological representation. On the other hand, we divide all the features into different types from multiple views and feed them to multiple classifiers instead of only one by combining different kernel matrices computed from different types of features optimally via multiple kernel learning. Moreover, we also apply feature selection method to choose the optimal feature subsets from redundant features for satisfying different datasets from different imaging devices. We implement our proposed classification system on three different datasets across more than 20 categories from phytoplankton to zooplankton. The experimental results validate that our system outperforms state-of-the-art systems for plankton image classification in terms of accuracy and robustness.

## Methods

**Fig. 1:**
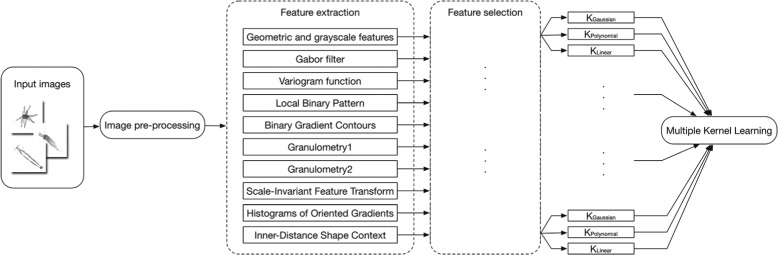
The framework of our proposed plankton image classification system

The automatic plankton image classification we proposed consists of five parts as follows: 1) image pre-processing, 2) feature extraction, 3) feature selection, 4) multiple kernel learning, and 5) evaluation. The framework is shown in Fig. 1.

### Image pre-processing

**Fig. 2:**
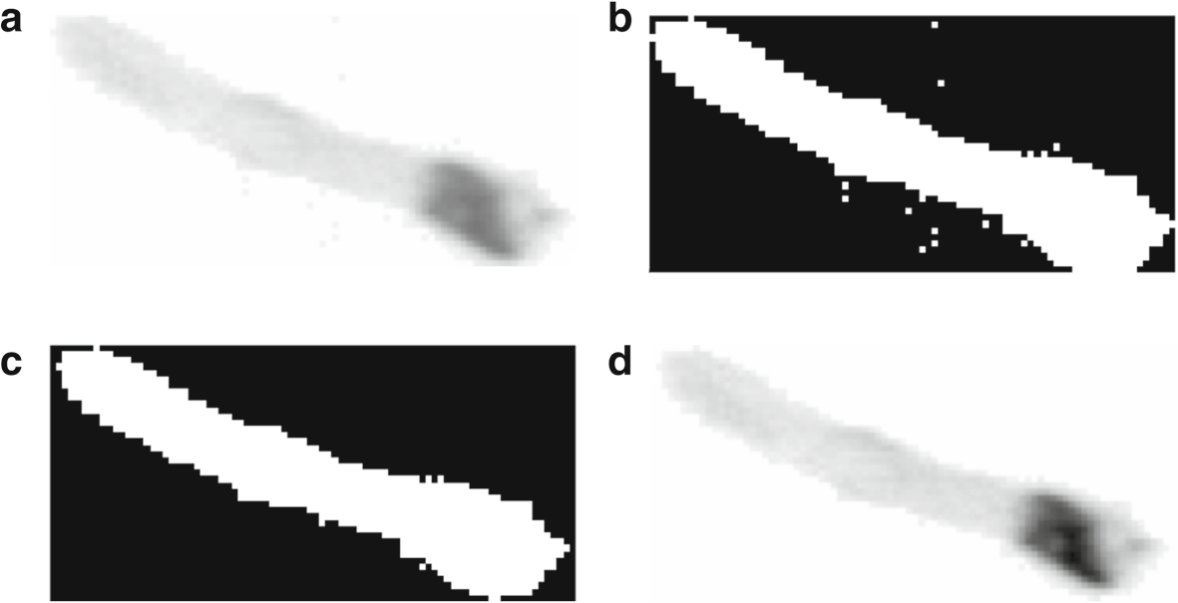
An example of image pre-processing. **a** Original captured plankton image. **b** Binarization. **c** Denoising. **d** Extraction

Images captured by (especially *in situ*) imaging devices mostly suffer from noise (Fig. 2 a). They may contain uninterested regions or unavoidable marine snow. To enhance the image quality and highlight the image features, we implement image pre-processing firstly to extract the plankton cells while reduce the noise such as marine snow in our system.

**Fig. 3:**
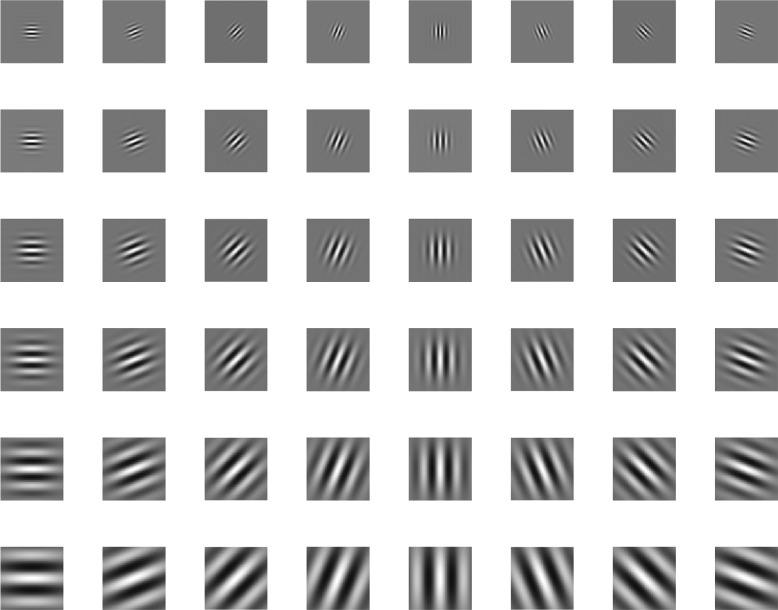
The Gabor filters with different parameters

The image pre-processing operation is the only part that may differ depending on the dataset, because the images acquired by different devices from different samples or locations are usually different in terms of noise and quality. But the objective and result of this operation are the same, that is, to extract the plankton cells with biomorphic characteristics from the original images. In our study, we focused on three different datasets acquired by IFCB, ZooScan, and *IS*IIS respectively, and designed the following unified steps: 1) binarization: convert the gray scale images to binary images (Fig. 2 b) based on threshold methods, 2) denoising: remove small connected regions (i. e., less than 5 pixels) due to the priori that they might not be plankton cells by morphological operations to obtain the binary mask (Fig. 2 c), and 3) extraction: extract the plankton cells (Fig. 2 d) from the original image using the denoised binary mask.

### Feature extraction

To obtain comprehensive characteristics of plankton, we extract various types of features in our classification system, including general features, which have been used for plankton classification previously, and robust features that are used extensively in object detection and recognition currently. The following will introduce our extracted features.

#### Geometric and grayscale features

Geometric features include size and shape measurements, such as area, circularity, elongation, convex rate, etc., and grayscale features include sum, mean, standard deviation, etc., and these features can describe the basic morphological characteristics of plankton and have been used in the previous study [17, 27, 29]. In our system, the geometric and grayscale features we applied consist of 43 elements represented by a 43-dimensiontal feature vector.

#### Texture features

Texture is one of the important characteristics used in plankton identification [17, 27]. In our system, we applied four method for texture feature extraction, including Gabor filter, variogram function, Local Binary Pattern (LBP), and Binary Gradient Contours (BGC).

**Gabor filter** Frequency and orientation representations of Gabor filters, which are similar to those of human visual system, are appropriate for texture representation [34]. In the spatial domain, a 2D Gabor filter is a Gaussian kernel function. The impulse response of these filters is created by convoluting a Gaussian function

1 $$  g(x,y)=\frac{1}{2\pi\sigma^{2}}e^{\left[-\frac{x^{2}+y^{2}}{2\sigma^{2}}+2\pi jF(x\cos\theta+y\sin\theta)\right]}  $$

where *θ* represents the orientation, *F* represents the center frequency, and *σ* is the standard deviation. Gabor filter is an essentially convolution of original image

2 $$  Q(x,y)=I(x,y)\ast g(x,y)  $$

where *I*(*x,y*) is the original image, *Q*(*x,y*) is the Gabor filter result. The mean value and standard deviation of Gabor filter result can be used to describe the texture feature

3 $$\begin{array}{@{}rcl@{}}  mean &=& \frac{\sum_{x=0}^{n-1}\sum_{y=0}^{m-1}Q(x,y)}{m\times n} \end{array} $$

4 $$\begin{array}{@{}rcl@{}} std &=& \sqrt{\frac{\sum_{x=0}^{n-1}\sum_{y=0}^{m-1}\left[Q(x,y)-mean\right]^{2}}{m\times n}} \end{array} $$

where *m,n* represent the size of image. A set of Gabor filters with different frequencies and orientations will be helpful for description of characteristics completely. In our system, we use Gabor filters with 6 kinds of frequencies and 8 kinds of orientations for plankton texture representation as shown in Fig. 3. Therefore, we obtained 48 mean values and standard deviation values to construct a 96-dimentional feature vector.

**Variogram function** The variogram, which is the basic function in geostatistics, is widely used for extraction of texture characteristics. The mathematical expression of variogram is

5 $$  \gamma(h)=\frac{1}{2N(h)}\sum_{i=1}^{N(h)}\left[I(x)-I(x+h)\right]^{2}  $$

where *h* is certain lag, *N*(*h*) is the number of experimental pairs, and *I*(*x*),*I*(*x*+*h*) are pixel values at *x,x*+*h*. In our system, we applied variogram *γ* to describe texture features.

**Fig. 4:**
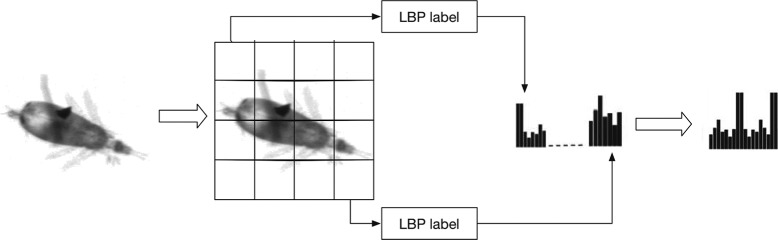
The LBP features

**Local binary pattern** LBP is a classical texture descriptor designed for classification and recognition, especially face recognition [35]. The basic idea of LBP is that two-dimensional surface textures can be described by local spatial patterns and gray scale contrast. The original LBP algorithm labels each pixel of image with 8-bit binary codes called LBP labels, which are obtained by the local structure (i.e., neighborhood) around the pixel. The histogram of these LBP labels can be used as texture descriptor. In our study, we improved the original LBP descriptor by segmenting the image into cells and then concatenating all the cell-based histograms as shown in Fig. 4, which can represent the part-based biomorphic features well.

**Binary gradient contours** BGC [36] relies on computing the gradient between pairs of pixels all along a closed path around the central pixel of a grayscale image patch. The most frequently used paths are single-loop, double-loop and triple-loop. And the binary gradient contours of single-loop are expressed as

6 $$\begin{array}{*{20}l}  g_{1}&=\left[ \begin{array}{c} s(I_{7}-I_{0})\\ s(I_{6}-I_{7})\\ s(I_{5}-I_{6})\\ s(I_{4}-I_{5})\\ s(I_{3}-I_{4})\\ s(I_{2}-I_{3})\\ s(I_{1}-I_{2})\\ s(I_{0}-I_{1}) \end{array}\right] \quad \text{where}\ s(x)\\&=\left\{\begin{array}{ll} 1 & x>0\\ 0 & x<0\end{array}\right., I_{k}\ \text{indicates neighbor pixel values.} \end{array} $$

Then all grayscale patterns can be mapped to the binary gradient contour value of single-loop by

7 $$  {}BGC1_{3\times 3}=w_{8}^{T}g_{1}-1\ \text{where}\ w_{j}^{T}\,=\,\left[ \!2^{j-1}\!\! \quad 2^{j-2}\!\!\! \quad \cdots\!\!\! \quad 2^{1}\! \quad 2^{0}\right]  $$

The texture is described by the histogram that quantify the occurrence of BGC value in images. In our system, we used the single-loop BGC descriptor.

#### Granulometric feature

Granulometry [37] is an approach to measure the size distribution of grains in binary image. It describes the particles range and distribution using a series of opening operators with structuring elements of increasing size

8 $$\begin{array}{@{}rcl@{}}  B\circ T & = & U\left\{T+x: T+x\subset B\right\} \end{array} $$

9 $$\begin{array}{@{}rcl@{}} \Psi_{\lambda}(B) & = & B\circ\lambda T \end{array} $$

where *B* denotes binary image, *Ψ*_*λ*_(*B*) denotes the result binary image, *T* is structuring elements, ∘ means opening operation and *λ* is the number of opening operation times. The granulometric size distribution of *B* is given by

10 $$  F_{B}(\lambda)=1-\frac{v(\Psi_{\lambda}(B))}{v(B)}  $$

where *v*(*B*) indicates the pixel number of grains. Therefore, granulometry can represent the multiscale shape feature of object. In our system, we used granulometry to describe the shape feature of plankton with two different setups: one is the size of elements increasing from 2 to 50 at interval of 4, and the other is the size of elements increasing from 5 to 60 at interval of 5.

#### Local features

Local features refer to patterns or distinct structures found in an image, such as points, edges, etc., and they can describe local image structures while handle scale changes, rotation as well as occlusion.

**Fig. 5:**
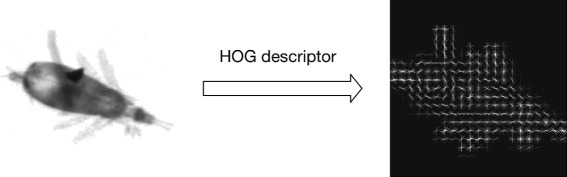
The HOG features

**Histograms of oriented gradients** HOG [38] counts occurrences of gradient orientation in localized portions of image. The main idea is that local object appearance and shape within an image can be described by the distribution of intensity gradients or edge directions, e.g., Fig. 5. In our system, every image is processed into square and resized to 256×256, then it is decomposed into 32×32 cells, and our HOG feature descriptor is constructed by the concatenation of the histograms of gradient directions of these cells.

**Fig. 6:**
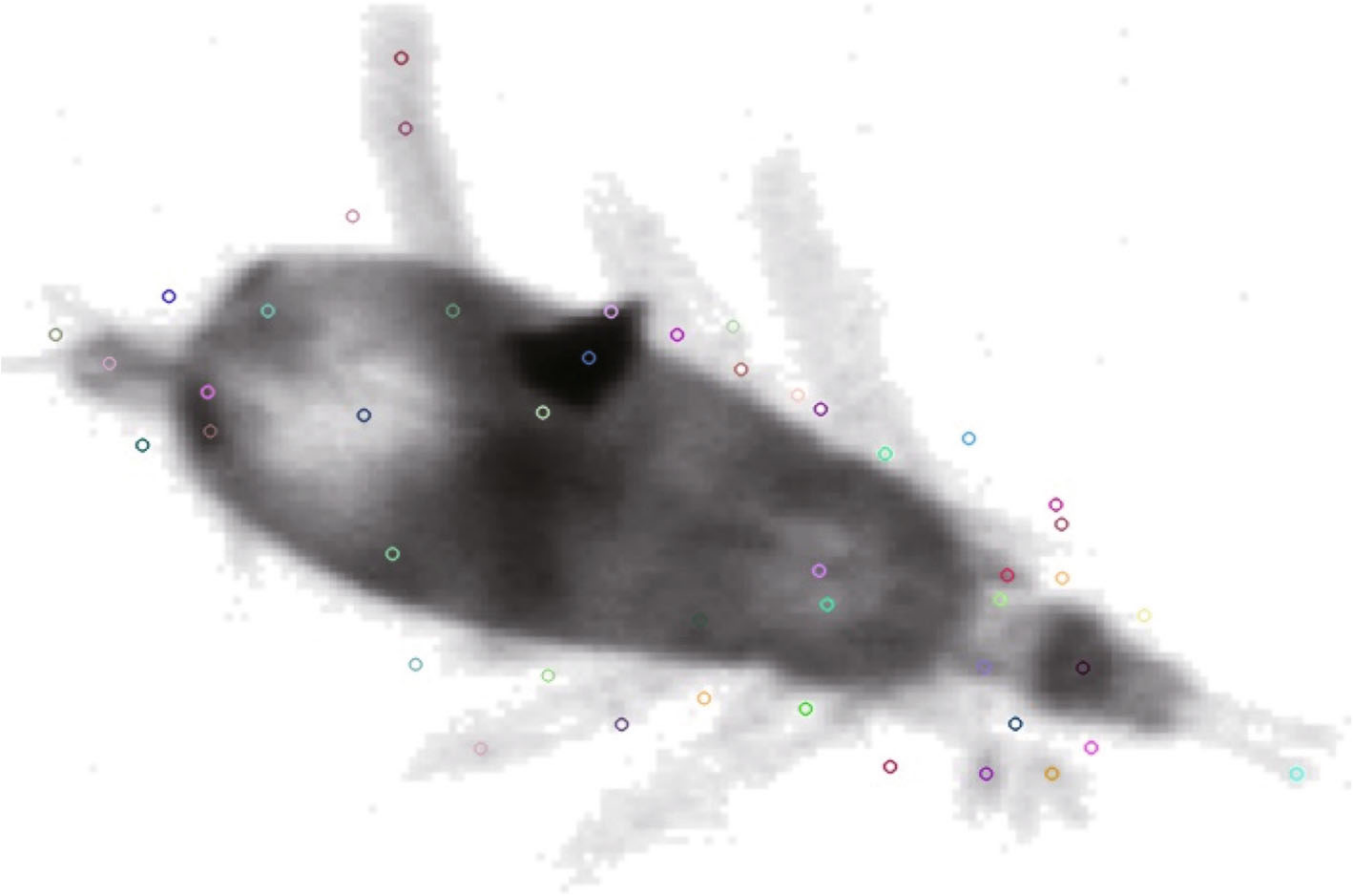
The keypoints of SIFT features

**Scale-Invariant feature transform** SIFT [39] is a well-known robust algorithm to detect and describe local features of image against scale and rotation changes by extracting keypoints (Fig. 6) and computing their descriptors, which has been widely used for object recognition, robotic mapping, video tracking, and so on. In image classification, SIFT usually integrates with bag-of-words (BoW) model to treat image features as words: first, use SIFT to extract keypoints of all images in dataset; second, divide all keypoints into groups by K-means clustering with codewords as the centers of learned clusters; then, the keypoints in an image can be mapped to a certain codeword through the clustering and an image can be represented by the *n*-bin histogram of the codewords. In our system, we set the number of clusters to 100, and every image is described by a 100-dimensional feature vector.

**Inner-Distance shape context** IDSC [40] is extended from Shape Context (SC) [41] designed for shape representation that describes the relative spatial distribution (distance and orientation) of landmark points around feature points. Given *n* sample points *P*={*p*_1_,⋯,*p*_*n*_} on a shape, the shape context at point *p*_*i*_ is defined as a histogram *h*_*i*_ of the relative coordinates of the remaining *n*−1 points

11 $$  h_{i}(k)=\#\left\{q\neq p_{i}: (q-p_{i})\in bin(k)\right\}  $$

where the bins uniformly divide the log-polar space. Shape context can be applied to shape matching by calculating the similarity between two shapes. The cost of matching two points *p*_*i*_,*q*_*j*_ is computed by

12 $$  C_{ij}=C(p_{i},q_{j})=\frac{1}{2}\sum_{k=1}^{K}\frac{\left[h_{i}(k)-h_{j}(k)\right]^{2}}{h_{i}(k)+h_{j}(k)}  $$

The matching *π* should minimize the match cost *H*(*π*) defined as

13 $$  H(\pi)=\sum_{i}C(p_{i},q_{\pi(i)})  $$

Once the best matching is found, the matching cost *H*(*π*) is the similarity measurement between shapes, that is, the shape distance. The shape context uses the Euclidean distance to measure the spatial relation between landmark points, which may cause less discriminability for complex shapes with articulations. The inner-distance, defined as the length of the shortest path within the shape boundary, is a natural way to solve this problem since it captures the shape structure better than Euclidean distance. In our system, we applied IDSC-based shape matching to describe shape features of plankton as follows: first, pick three images from each category of dataset and manually extract their shapes as templates; second, use IDSC to match shape of every image with templates and compute the shape distances between them; then, obtain the shape distances as the feature vector for shape representation.

### Feature selection

In machine learning, feature selection is an important process of selecting the optimal features for classification, because the redundant features can suppress the performance of classifier. Besides, feature selection can reduce training time and improve the efficiency, especially for high-dimensional features. Thus, we applied wrapper method [42] for feature selection to choose the optimal features from aforementioned various high-dimensional features for performance improvement. In our system, we divided all features into ten types (Fig. 1), and we applied feature selection on each type of features to choose the optimal features respectively.

### Multiple kernel learning

Multiple kernel learning (MKL) is a set of machine learning methods that use a predefined set of kernels and learn an optimal linear or non-linear combination of multiple kernels. It can be applied to select for an optimal kernel and parameters, and combine different types of features. Recently, MKL has received great attention and been used in many recognition and classification applications, such as visual object recognition [43] and hyperspectral image classification [44]. MKL aims to learn a function of the form

14 $$  f(x)=\sum_{i=1}^{l}\alpha_{i}y_{i}f_{\eta}\left(\left\{K_{m}\left.\left(x_{i}^{m},x_{j}^{m}\right)\right)\right\}_{m=1}^{M}\right)+b  $$

with *M* multiple kernels instead of a single one

15 $$  K_{\eta}(x_{i},x_{j})=f_{\eta}\left(\left\{K_{m}\left(x_{i}^{m},x_{j}^{m}\right)\right\}_{m=1}^{M}\right)  $$

where *f*_*η*_ is the combination function of kernels, and it can be a linear or non-linear function.

According to the functional form of combination, the existing MKL algorithms can be grouped into three basic categories [31]: 1) linear combination methods, such as SimpleMKL [45], GLMKL [46], 2) nonlinear combination methods, such as GMKL [47], NLMKL [48], and 3) data-dependent combination methods, such as LMKL [49]. Gönen and Alpaydin [31] also performed experiments on real datasets for comparison of existing MKL algorithms and gave an overall comparison between algorithms in terms of misclassification error. It concluded that using multiple kernels is better than using a single one and nonlinear or data-dependent combination seem more promising. Based on their experiments and our analysis, in our system, we chose NLMKL [48], a nonlinear combination of kernels, as MKL method to combine multiple extracted plankton features. NLMKL is based on a polynomial combination of base kernels shown as

16 $$  K_{\eta}\left(x_{i},x_{j}\right)=\sum_{k_{1}+\cdots+k_{p}}\eta_{1}^{k_{1}}\cdots\eta_{p}^{k_{p}}K_{1}^{k_{1}}\cdots K_{p}^{k_{p}}  $$

We used NLMKL to combine three kernel functions, Gaussian kernel, polynomial kernel, and linear kernel, on each type of features (Fig. 1).

### Evaluation

A confusion matrix (Table 1) is a table containing information about actual and predicted classifications, so that it can be used to evaluate the performance of classification systems. Each column of confusion matrix represents the samples in a predicted class while each row represents the samples in an actual class. And the diagonal of the matrix represents correct identifications of samples. Several measures can be derived from a confusion matrix, for instance, true positive rate (TPR, also called recall), false negative rate (FNR), false positive rate (FPR), true negative rate (TNR), positive predictive value (PPV, also called precision). In our system, we use *Recall* and *Precision* (actually 1−*Precision*, means error rate) to evaluate the performance of classification

17 $$ {}\begin{aligned} TPR (\text{or}Recall\text{or }R)\quad\; &= \frac{\sum True\ positive}{\sum Condition\ positive} \end{aligned}  $$

**Table 1:** Confusion matrix

		Predicted condition	
	Total population	Prediction positive	Prediction negative
True condition	Condition positive	True positive (TP)	False negative (FN)
	Condition negative	False positive (FP)	True negative (TN)

18 $$ {}\begin{aligned} PPV (\text{or}Precision\text{or }P) &= \frac{\sum True\ positive}{\sum Predicted\ condition\ positive} \end{aligned}  $$

where *True* *positive* is the number of samples correctly predicted, and *Condition* *positive* is total number of actual samples. And higher *R* with lower 1−*P* will give better classification performance. Then we use *F*_*measure*_ (higher) that combines precision and recall with harmonic mean to evaluate the performance (better) of classification system

19 $$  F_{measure}=2\times\frac{P\times R}{P+R}  $$

## Results

To illustrate our proposed plankton image classification system, we perform three experiments on three publicly available datasets collected by different imaging devices in different locations with more than 20 categories covering phytoplankton and zooplankton.

### Datasets

#### WHOI dataset

**Fig. 7:**
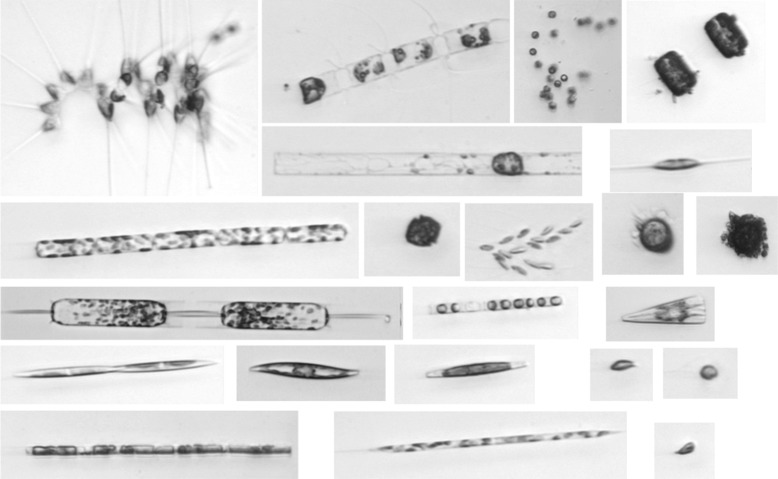
Image examples from WHOI dataset

This dataset was collected with IFCB [6] from Woods Hole Harbor water. All sampling was done between late fall and early spring in 2004 and 2005 [17] and can be accessed at: http://onlinelibrary.wiley.com/doi/10.4319/lom.2007.5.204/full. It contains 6600 images with distribution across 22 categories (Fig. 7), and most categories are phytoplankton taxa at the genus level, among which 16 categories are diatoms: *Asterionellopsis* spp., *Chaetoceros* spp., *Cylindrotheca* spp., *Cerataulina* spp. plus the morphologically similar species of *Dactyliosolen* such as *D. fragilissimus*, other species of *Dactyliosolen* morphologically similar to *D. blavyanus*, *Dinobryon* spp., *Ditylum* spp., *Euglena* spp. plus other euglenoids, *Guinardia* spp., *Licmophora* spp., *Phaeocystis* spp., *Pleurosigma* spp., *Pseudonitzschia* spp., *Rhizosolenia* spp. and rare cases of *Proboscia* spp., *Skeletonema* spp., *Thalassiosira* spp. and similar centric diatoms; the remaining categories are mixtures of morphologically similar particles and cell types: ciliates, detritus, dinoflagellates greater than 20*μ* *m*, nanoflagellates, other cells less than 20*μ* *m*, and other single-celled pennate diatoms. The images were split between training and testing sets of equal size, and each set contains 22 categories with 150 individual images in each. Accordingly, in our experiments, we used the training set for learning and the testing set to assess the performance of the classification system.

#### ZooScan dataset

**Fig. 8:**
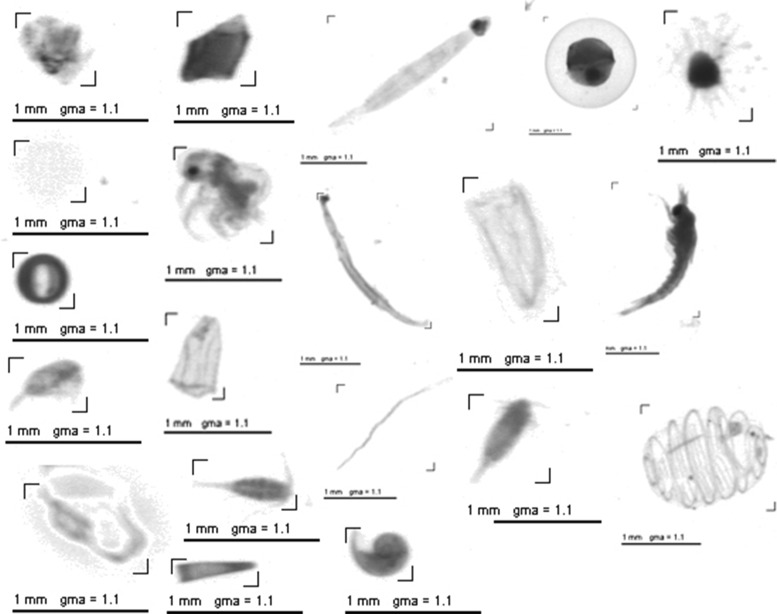
Image examples from ZooScan dataset

**Fig. 9:**
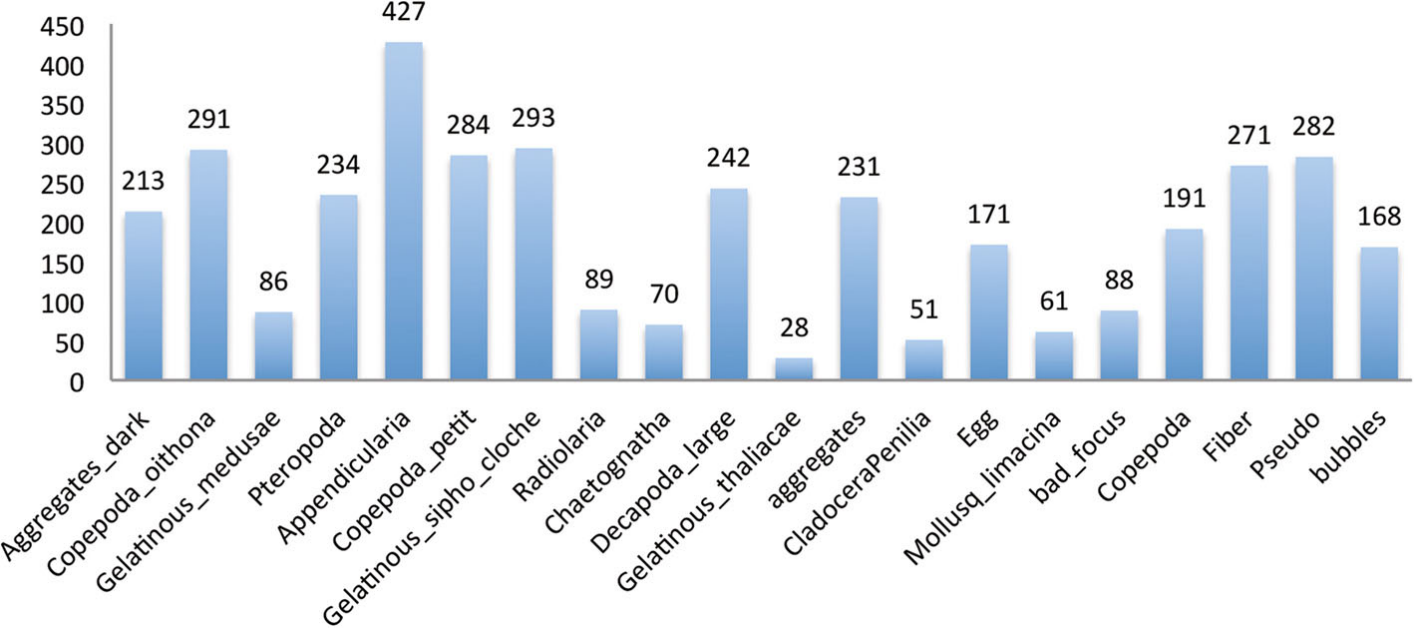
The number of images per category in ZooScan dataset

This dataset was collected by the ZooScan system (http://www.zooscan.com) with a series of samples from the Bay of Villefranche-sur-mer, France between 22 August 2007 and 8 October 2008 [8]. It contains 3771 zooplankton images of 20 categories (Fig. 8), among which 14 categories are zooplankton: *Limacina*, Pteropoda, Penilia, *Oithona*, Poecilostomatoida, other species of Copepoda, Decapoda, Appendicularia, Thaliaca, Chaetognatha, Radiolaria, Calycophorae, other species of Medusae, and eggs of zooplankton; the remaining categories are non-zooplankton: bubble, fiber, aggregates, dark aggregates, pseudoplankton, and images with bad focus. The number of images in each category is different as shown in Fig. 9. In our experiments on this dataset, we used 2-fold cross validation to evaluate the performance of the classification system.

#### Kaggle dataset

**Fig. 10:**
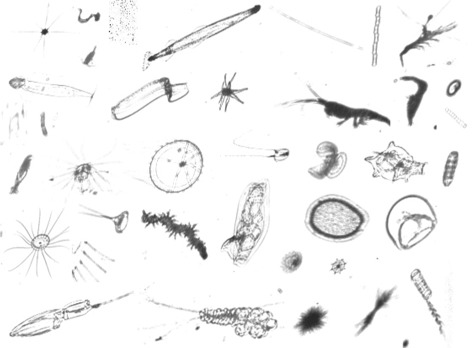
Image examples from Kaggle dataset

**Fig. 11:**
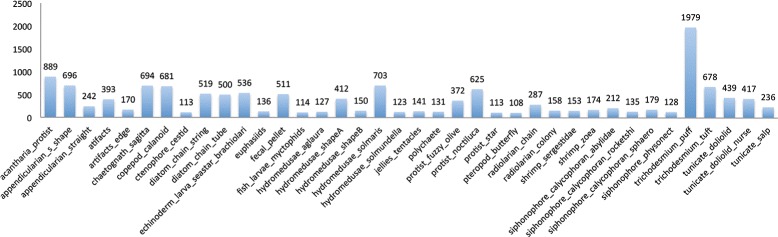
The number of images per category in Kaggle dataset

This dataset was collected between May-June 2014 in the Straits of Florida using *IS*IIS [7], and was first published on Kaggle (https://www.kaggle.com/c/datasciencebowl) with data provided by the Hatfield Marine Science Center at Oregon State University. It consists of 121 categories ranging from the smallest single-celled protists to copepods, larval fish, and larger jellies. In our experiments, we chose 38 categories (Fig. 10) with more than 100 individual images in each (Fig. 11) to construct a new dataset, among which 35 categories are plankton and 3 categories are non-plankton including atifacts, atifacts edge and fecal pellet. The constructed dataset contains 28748 images, and we used 5-fold cross validation to evaluate the performance of the classification system.

### Experiments

We designed three experiments on above three datasets for evaluation of our classification system: first, we built the baseline system for benchmarking state-of-the-art plankton image classification systems; then, we used SVM with three kernels to compare our extracted features with the baseline; at last, we applied NLMKL on our extracted features to compare our final system with SVM system.

#### Baseline

To illustrate the performance of our proposed system, we should first build a baseline system to benchmark state-of-the-art plankton image classification systems. The baseline system is built as follows: 1) feature extraction: extract the 210 features used by Sosik and Olson [17] and the 53 features used in ZooScan system [8] to construct a 263-dimensional feature vector, 2) feature selection: apply the feature selection algorithm used in [17] to choose optimal features while remove redundant features and obtain 99, 80, and 100 dimensions of features for WHOI, ZooScan, and Kaggle datasets respectively, and 3) classifier: use SVM with Gaussian kernel to train the classifier and select the optimal *C* and gamma by searching over the grid of appropriate parameters.

**Fig. 12:**
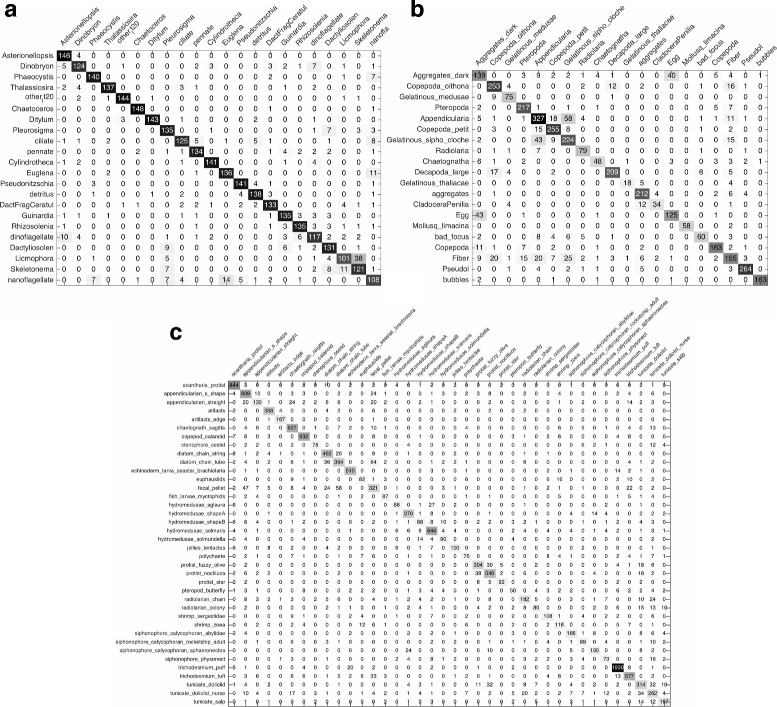
Confusion matrices of the baseline system. **a** WHOI dataset. **b** ZooScan dataset. **c** Kaggle dataset

The classification results of baseline system on three datasets are listed in Table 2 and the confusion matrices are shown in Fig. 12. It can be seen that the best classification performance of three datasets are 88.27% *Recall* with 11.63% 1−*Precision*, 80.6% *Recall* with 16.3% 1−*Precision*, and 75.36% *Recall* with 21.49% 1−*Precision*, respectively. The performances on WHOI dataset and ZooScan dataset are a little better than that of Sosik and Olson [17] with 88% overall accuracy and ZooScan system [8] of 78% *Recall* with 19% 1−*Precision*. Obviously, the system, which combines their methods, has better performance. Therefore, this baseline can be used as the benchmark for performance evaluation of our proposed system.

**Table 2:** The classification results of the baseline system

	WHOI dataset	ZooScan dataset	Kaggle dataset
*R*	88.27%	80.6%	75.36%
1−*P*	11.63%	16.3%	21.49%
*F*_*measure*_	0.883	0.821	0.769

#### Comparison of features

This experiment is designed for performance evaluation of our extracted features, and it is implemented as follows: 1) feature extraction: extract all the features presented in “Feature extraction” section, 2) feature grouping: group all the features into 10 types that are geometric and grayscale features, Gabor features, variogram features, LBP features, BGC features, granulometry1 features, granulometry2 features, SIFT features, HOG features, and IDSC features, 3) feature selection: employ feature selection method presented in “Feature selection” section on each type of features separately and concatenate the output features to obtain 139, 148, and 233 dimensions of features for WHOI, ZooScan, and Kaggle datasets respectively, and 4) classifiers: use SVM with Gaussian, polynomial, and linear kernels to train the classifiers.

**Fig. 13:**
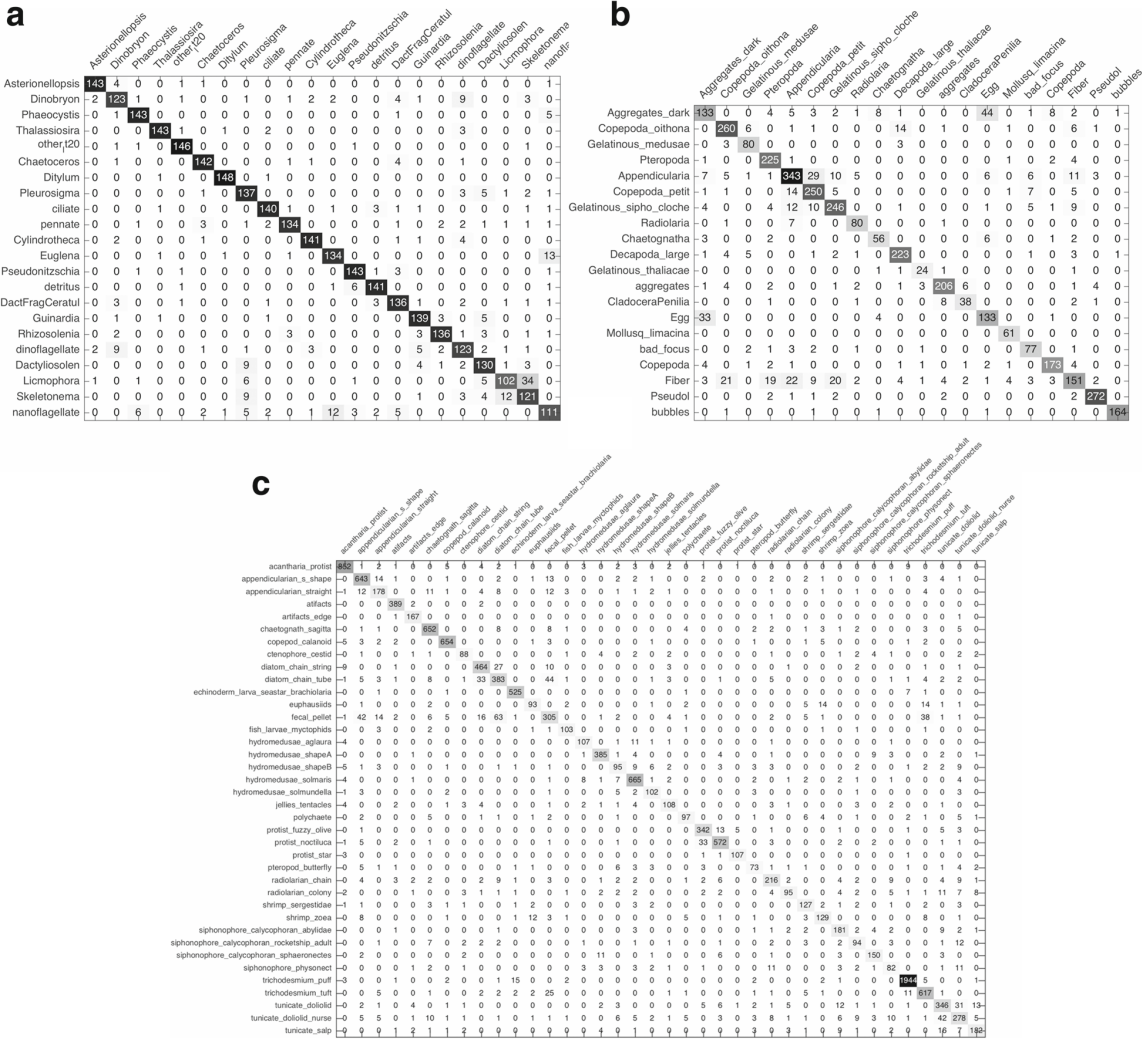
Confusion matrices of multiple view features using SVM. **a** WHOI dataset. **b** ZooScan dataset. **c** Kaggle dataset

The experimental results on three datasets are listed in Table 3 and the confusion matrices are shown in Fig. 13. It can be seen that the best classification performance with highest *F*_*measure*_ on WHOI dataset is 89.57% *Recall* with 10.3% 1−*Precision* (Gaussian kernel and *C*=100), which provides 1.3 percentage points better *Recall* than baseline, and the best classification performance on ZooScan and Kaggle datasets are 85.39% *Recall* with 13.2% 1−*Precision* and 82.41% *Recall* with 16.33% 1−*Precision* respectively (Gaussian kernel and *C*=10), which provide 4.79 and 7.05 percentage points better *Recall* than baseline. Consequently, the features we extracted are able to show more complete description of plankton characteristics and improve the performance of classification.

**Table 3:** The classification results of multiple view features using SVM

Datasets	C	Gaussian			Polynomial			Linear		
		*R*	1−*P*	*F*_*measure*_	*R*	1−*P*	*F*_*measure*_	*R*	1−*P*	*F*_*measure*_
WHOI	1	84%	15.43%	0.843	88.97%	10.95%	0.89	86.45%	13.41%	0.865
	10	88.94%	11%	0.89	89.45%	10.47%	0.895	88.12%	11.78%	0.882
	100	**89.57%**	**10.3%**	**0.896**	88.42%	11.46%	0.885	86.33%	13.59%	0.864
ZooScan	1	79.65%	16.06%	0.817	82.45%	15.99%	0.832	79.91%	18.14%	0.809
	10	**85.39%**	**13.2%**	**0.861**	84.14%	15.22%	0.845	85.52%	16.01%	0.847
	100	84.87%	13.62%	0.856	83.04%	16.02%	0.835	82.27%	18.23%	0.82
Kaggle	1	77.26%	18.96%	0.791	77.48%	19.6%	0.789	71.32%	25.09%	0.731
	10	**82.41%**	**16.33%**	**0.83**	80.7%	18.08%	0.813	78.44%	20.63%	0.789
	100	82.09%	18.89%	0.816	79.01%	19.73%	0.796	78.1%	22.05%	0.78

The entries in boldface indicate the best classification results with the highest *F*_*measure*_

#### Comparison of systems

This experiment is designed for performance evaluation of our proposed system, and it is implemented as follows: 1) feature extraction, 2) feature grouping, and 3) feature selection are the same as the “Comparison of features” experiment, and 4) classifiers: use NLMKL to combine various types of features with one kind of kernel and three kinds of kernel to train the classifiers respectively.

**Fig. 14:**
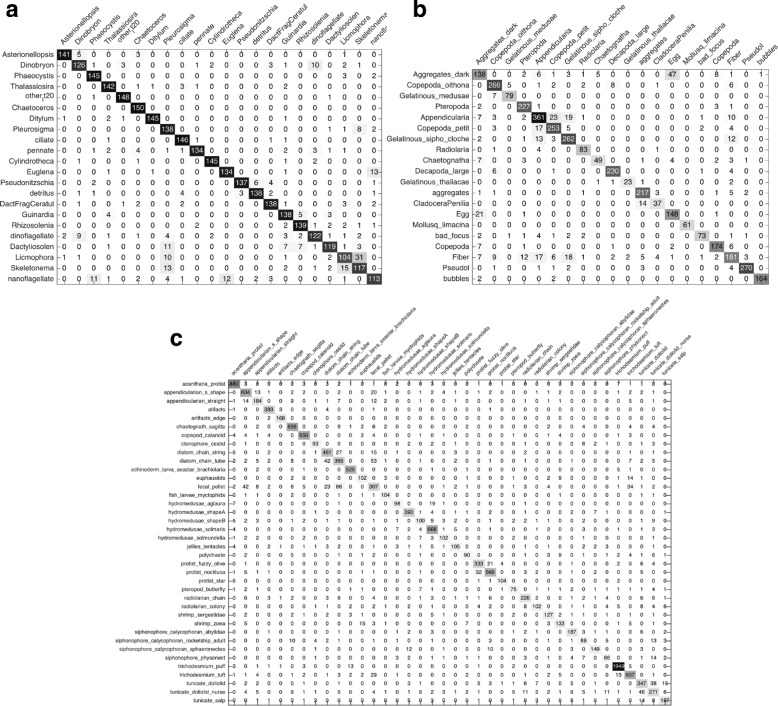
Confusion matrices of multiple view features using MKL with one kind of kernel. **a** WHOI dataset. **b** ZooScan dataset. **c** Kaggle dataset

The experimental results on three datasets are listed in Table 4 (one kind of kernel) and Table 5 (three kinds of kernel), and the confusion matrices are shown in Fig. 14. It can be seen that the best classification performance with highest *F*_*measure*_ of MKL system with one kind of kernel on three datasets are 89.67% *Recall* with 10.16% 1−*Precision* (polynomial kernel and *C*=10), 86.6% *Recall* with 9.92% 1−*Precision* (Gaussian kernel and *C*=100), and 82.6% *Recall* with 15.62% 1−*Precision* (polynomial kernel and *C*=10), which provide 0.1, 1.21, and 0.19 percentage points better *Recall* than SVM system, while the best classification performance of MKL system with three kinds of kernel on three datasets are 90% *Recall* with 9.91% 1−*Precision*, 88.34% *Recall* with 9.58% 1−*Precision*, and 83.67% *Recall* with 14.49% 1−*Precision*, which provide 0.33, 1.74, and 1.07 percentage points better *Recall* than the MKL system with one kind of kernel as well as 1.73, 7.74, and 8.31 percentage points better *Recall* than baseline, respectively. The results validate that MKL is more effective than SVM for classification, and combining more kinds of kernel can provide better performance than only one kind of kernel.

**Table 4:** The classification results of multiple view features using MKL with one kind of kernel

Datasets	C	Gaussian			Polynomial			Linear		
		*R*	1−*P*	*F*_*measure*_	*R*	1−*P*	*F*_*measure*_	*R*	1−*P*	*F*_*measure*_
WHOI	1	88.48%	11.35%	0.886	89.58%	10.21%	0.897	88.55%	11.18%	0.887
	10	88.75%	11.04%	0.889	**89.67%**	**10.16%**	**0.898**	89.12%	10.65%	0.892
	100	88.58%	11.2%	0.887	89.39%	10.44%	0.895	88.42%	11.41%	0.885
ZooScan	1	83.32%	11.74%	0.857	83.94%	12.61%	0.856	81.78%	15.53%	0.831
	10	86.26%	10.01%	0.881	86.74%	11.76%	0.875	83.98%	15.28%	0.843
	100	**86.6%**	**9.92%**	**0.883**	86.79%	11.63%	0.876	84.86%	19.13%	0.828
Kaggle	1	78.46%	17.41%	0.805	80.39%	16.76%	0.818	78.09%	19.24%	0.794
	10	82.95%	16.42%	0.833	**82.6%**	**15.62%**	**0.835**	81.32%	17.66%	0.818
	100	82.97%	16.84%	0.831	82.11%	15.82%	0.831	79.68%	19.1%	0.803

The entries in boldface indicate the best classification results with the highest *F*_*measure*_

**Table 5:** The classification results of multiple view features using MKL with three kinds of kernel

Datasets	C	Gaussian+Polynomial+Linear		
		*R*	1−*P*	*F*_*measure*_
WHOI	1	89.64%	10.17%	0.897
	10	89.88%	10.03%	0.899
	100	**90%**	**9.91%**	**0.9**
ZooScan	1	85.42%	11.38%	0.87
	10	**88.34%**	**9.58%**	**0.894**
	100	88.31%	9.81%	0.892
Kaggle	1	80.3%	16.12%	0.82
	10	**83.67%**	**14.49%**	**0.846**
	100	83.46%	14.88%	0.843

The entries in boldface indicate the best classification results with the highest *F*_*measure*_

## Discussion

An automated plankton classification system for abundance estimation of different plankton categories is presented in our work. The goal of our work points to develop a system that can be widely used for classification of phytoplankton and zooplankton with higher accuracy for ecological studies. With this aim, we proposed an automatic system for plankton classification combining various types of features from different views using MKL, so that it can make better use of information from multiple views and improve the classification performance in terms of accuracy.

The current study of plankton image classification system mainly focuses on one specific imaging device with several categories of phytoplankton or zooplankton, so that it’s hard to have both high accuracy and wide suitability. In order to broaden the application scope and improve the performance of plankton image classification system, we developed the system with threefold contributions: 1) we extracted features from all conceivable views to describe plankton morphological characteristics more completely and comprehensively, 2) we used MKL to combine different views of features for better “understanding” of extracted information, and 3) we combined linear and nonlinear kernels for MKL to obtain better performance than state-of-the-art systems.

In order to evaluate the performance of our proposed system for plankton image classification, three different datasets were collected and constructed while three experiments were designed and implemented in our study. The three datasets, named WHOI, ZooScan and Kaggle, were collected by three different imaging devices, i.e., IFCB, ZooScan, and *IS*IIS, respectively. The images were sampled in different locations and cover wide categories more than 20 from phytoplankton to zooplankton. The baseline experiment was designed and implemented on the three datasets with state-of-the-art plankton image classification methods to give a benchmark for evaluation. The comparison experiments of features and systems were designed to validate the effectiveness and robustness of features and systems respectively. And the experimental results show that our multiple view features performs better than state-of-the-art and our MKL system combing these multiple view features performs best in all the experiments.

Tables 2 and 3 illustrate the accuracy of the first and second experiments. In comparison, it can be seen that our proposed multiple view features are helpful to improve the plankton classification accuracy, since that the best classification performance of *Recall* on three datasets increase by 1.3% (WHOI), 4.79% (ZooScan), and 7.05% (Kaggle), and meanwhile the corresponding error rates drop by 1.33% (WHOI), 3.1% (ZooScan), and 5.16% (Kaggle), respectively. And by comparing the confusion matrices shown in Figs. 12 and 13, we can find that the classification accuracy of 14 categories in WHOI dataset, 16 categories in ZooScan dataset, and 36 categories in Kaggle dataset are improved in the second experiment. These results show that our proposed multiple view features can describe the plankton morphological characteristics more completely and comprehensively, for example, as shown in Fig. 12, 58 images of “Appendicularia” category in ZooScan dataset are incorrectly classified as “Gelatinous_sipho_cloche” in the baseline experiment, but this misclassified number drops from 58 to 10 in the second experiment using our multiple view features shown in Fig. 13.

With the same multiple view features, the second and third experiments use different machine learning strategies for classification. By comparing Table 3 with Table 4, we can observe that MKL provides better performance than SVM. NLMKL with one kind of kernel in the third experiment provides 0.1 (WHOI), 1.21 (ZooScan), and 0.19 (Kaggle) percentage points better *Recall* than SVM, and meanwhile the corresponding error rates drop by 0.14% (WHOI), 3.28% (ZooScan), and 0.71% (Kaggle), respectively. And by comparing the confusion matrices shown in Figs. 13 and 14, there are 11 categories in WHOI dataset, all 20 categories in ZooScan dataset, and 21 categories in Kaggle dataset having higher accuracy in the third experiment. By comparing Table 4 with Table 5, we can find that MKL with more kernels performs better than only one. In other words, MKL, which optimally combines different kernel matrices computed from multiple types of features with multiple kernel functions, can make better use of information of each type of features and improve the classification performance significantly. Not surprisingly, MKL has been proven to be a useful tool for feature combination to enhance the discrimination power of classifiers, for example, Althloothi et al. [50] improved the performance of human activity recognition by combining two sets of features using MKL, and Luo et al. [51] proved the effectiveness of MKL for feature combination.

Our overall results compare favorably with previous results. Our proposed plankton image classification system has a classification performance of 90% *Recall* on WHOI dataset, 88.34% *Recall* on ZooScan dataset, and 83.67% *Recall* on Kaggle dataset, respectively, and meanwhile the corresponding error rates have the similar degree of decline. It is effective for situations like these three datasets. By comparing the confusion matrices shown in Figs. 12 and 14, it can be seen that most categories of the three datasets have higher accuracy using our system than baseline with state-of-the-art systems. Based on these comparisons, we can conclude that multiple view features combination using MKL enhances the performance of classification and accommodates a wide variety of plankton categories while gives significantly better accuracy.

There is one important issue that we should discuss in this paper: deep learning for plankton image classification. Deep learning [52], also known as deep neural network, has been drawn more and more attention these years because of its dramatically performance on large-scale visual recognition and classification tasks. It attempts to model high level abstractions in data in order to learn representations of data end-to-end. This technology has also been used in plankton image classification recently [53–55]. However, currently, deep learning can perform good only when fed with enough human labeled data. That’s why it is strongly related to the word “large-scale”. With the development of *in situ* underwater microscopy imaging technology, it’s easy to collect thousands and thousands of plankton images in a short time (such as one day), but it’s still very hard to label all of them by experts because it needs very professional knowledge to identify them. Therefore, it would be very useful to build an end-to-end learning system with very less labeled data, which is also an important future work of deep learning field. The system proposed in this paper doesn’t need large-scale data and performs good on classification for more than 20 categories (but less than 50 categories), which is more suitable for the current actual applications of ecological studies.

## Conclusions

In this paper, we propose an automatic plankton image classification system combining multiple view features using multiple kernel learning. In our system, multiple view features, including general features and robust features, are extracted for better describing the morphological characteristics of plankton. And different types of features are combined via NLMKL using three kernel functions (linear, polynomial and Gaussian kernel functions) in order to use information of features better and achieve a higher classification rate. Our experimental results on three different datasets show the significance of the extracted multiple view features and MKL in plankton image classification. The main limitation of our system is lack of ability to work well with terribly imbalanced datasets, and we wish to leave this part in our future work.

To encourage future work, we make all the source code and datasets available in the GitHub repository: https://github.com/zhenglab/PlanktonMKL.

## Abbreviations

ADIAC: Automatic diatom identification and classification
ANN: Artificial neural network
BGC: Binary gradient contours
BoW: Bag-of-words
COM: Co-occurrence matrices
DiCANN: Dinoflagellate categorisation by artificial neural network
DT: Decision tree
FNR: False negative rate
FPR: False positive rate
GLMKL: Group Lasso-based MKL
GMKL: Generalized MKL
HMC: Hierarchical multi-label classification
HOG: Histograms of oriented gradients
IDSC: Inner-distance shape context
IFCB: Imaging FlowCytoBot
*IS*IIS: *In Situ* Ichthyoplankton imaging system
kNN: k Nearest neighbor
LBP: Local binary pattern
LDA: Linear discriminant analysis
LMKL: Localized MKL
LVQ: Learning vector quantization
MKL: Multiple kernel learning
NLMKL: Non-Linear MKL
NMDEE: Normalized multilevel dominant Eigenvector estimation
NN: Neural network
PPV: Positive predictive value
RF: Random forest
RPT: Recursive partitioning tree
SC: Shape context
SIPPER: Shadowed image particle profiling evaluation recorder
SIFT: Scale-invariant feature transform
SPC: Scripps plankton camera
SVM: Support vector machine
TNR: True negative rate
TPR: True positive rate
UVP: Underwater video profiler
VPR: Video plankton recorder
ZOOVIS: ZOOplankton VIsualization system
